# Synthesis of 6,12-Disubstituted Methanodibenzo[*b*,*f*][1,5]dioxocins: Pyrrolidine Catalyzed Self-Condensation of 2′-Hydroxyacetophenones

**DOI:** 10.3390/molecules24132405

**Published:** 2019-06-29

**Authors:** Benedicta Assoah, Vesa Riihonen, João R. Vale, Arto Valkonen, Nuno R. Candeias

**Affiliations:** 1 Faculty of Engineering and Natural Sciences, Tampere University, Korkeakoulunkatu 8, 33101 Tampere, Finland; 2 Instituto de Investigação do Medicamento (iMed.ULisboa), Faculdade de Farmácia, Universidade de Lisboa, Av. Prof. Gama Pinto, 1649-003 Lisboa, Portugal; 3 Department of Chemistry, University of Jyvaskyla, P.O. Box 35, 40014 Jyväskylä, Finland

**Keywords:** enamine, 1,5-dioxocin, self-condensation, 2′-hydroxyacetophenone

## Abstract

The preparation of unprecedented 6,12-disubstituted methanodibenzo[*b*,*f*][1,5]dioxocins from pyrrolidine catalyzed self-condensation of 2′-hydroxyacetophenones is herein described. This method provides easy access to this highly bridged complex core, resulting in construction of two C–O and two C–C bonds, a methylene bridge and two quaternary centers in a single step. The intricate methanodibenzo[*b*,*f*][1,5]dioxocin compounds were obtained in up to moderate yields after optimization of the reaction conditions concerning solvent, reaction times and the use of additives. Several halide substituted methanodibenzo[*b*,*f*][1,5]dioxocins could be prepared from correspondent 2′-hydroxyacetophenones.

## 1. Introduction

Heterocyclic compounds possess fascinating complex structural architecture and are key structural motifs in an array of biologically active natural products and pharmaceutically active compounds [1], which drives the development of improved and new synthetic methodologies [2,3,4,5,6,7,8,9]. In recent years, cascade reactions [10,11,12,13,14,15,16] and bio-inspired technologies [17,18,19,20,21,22,23] have been employed to access structural complexity in compounds of biological importance. 

**Scheme 1:**
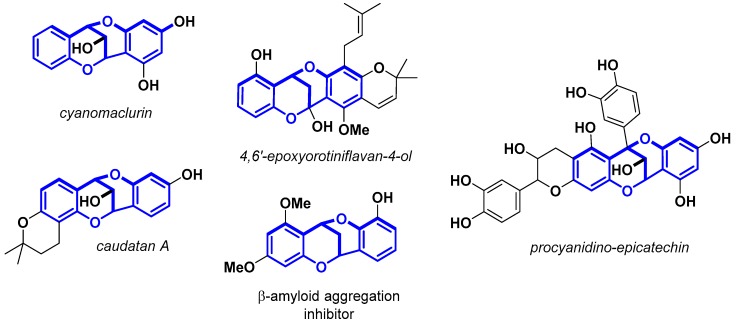
Selected reported biologically relevant methanodibenzo[*b*,*f*][1,5]dioxocins.

Methanodibenzo[*b*,*f*][1,5]dioxocin is a highly strained bridged polycyclic skeleton present in numerous biologically active natural products and pharmaceuticals (Scheme 1). Some of these natural products exhibit inhibition against β-amyloid aggregation, antibacterial activity, potent anti- methicillin-resistant staphylococcus aureus (MRSA) activity, etc. [24,25,26,27,28,29,30,31,32,33,34,35]. After Nair et al. [29] reported that cyanomaclurin, a compound isolated from the heartwood of *Artocarpus integrifolia* (jackwood) [36], possessed a methanodibenzo[*b*,*f*][1,5]dioxocin core, considerable attention was paid to its construction. Hennis and co-workers obtained the neutral 6H,12H-6,12-methanodibenzo[*b*,*f*][1,5]dioxocin and its derivatives after a condensation reaction of either *o*-vinylphenol or *o*-coumaric acid and salicylaldehyde under acidic conditions [37,38]. 

**Scheme 2:**
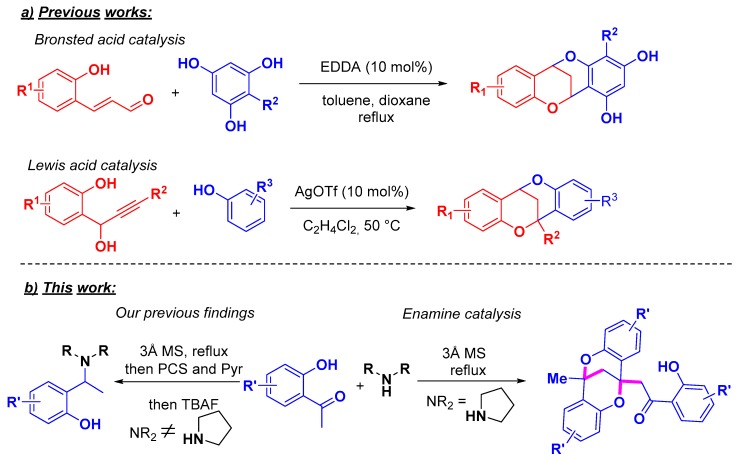
Previously reported catalytic methods for the synthesis of methanodibenzo[*b*,*f*][1,5]dioxocins (**a**) and our enamine catalyzed approach (**b**).

The catalytic preparation of the methanodibenzo[*b*,*f*][1,5]dioxocin core has been recently explored (Scheme 2a). A bioinspired cascade sequence of ethylenediaminediacetic acid (EDDA)-catalyzed olefin isomerization/hemiacetalization/dehydration/[3+3]-type cycloaddition driven by an in situ generated chromenylium intermediate has been reported by Liu et al. in the synthesis of methanodibenzo[*b*,*f*][1,5]dioxocin flavonoids from phloroglucinol and 2-hydroxycinnamalaldehyde [39]. Du and co-workers explored *o*-quinone methide and electron rich phenols as reactants in a silver triflate catalyzed tandem process for the construction of such a core. The formation of alkynyl *o*-quinone methide catalyzed by silver triflate triggers the following intermolecular cascade: 1,4-conjugate addition/6-endo cyclization/1,3-aryl shift/intramolecular 1,4-conjugate addition, providing the 2-substituted methanodibenzo[*b*,*f*][1,5]dioxocin [40]. Notwithstanding these recent developments, more synthetic strategies to access this intricate polycyclic ring system are worth pursuing as accessing [1,5]-dioxocins with different substituents in the bridgehead carbons is still an open issue. Likely due to the limited synthetic methodologies available, the methanodibenzo[*b*,*f*][1,5]dioxocin motif has received little attention as a structural scaffold in drug design. 

In our previous endeavor towards the development of a reductive amination protocol for the synthesis of tertiary alkylphenolmethyl amines [41] using a recently developed pinacol-derived chlorohydrosilane (PCS) [42], we were intrigued by the absence of the desired reductive amination product under our hydrosilylation conditions with 2′-hydroxyacetophenone and pyrrolidine (Scheme 2b). Despite the successful use of such a protocol for the synthesis of several tertiary alkylphenolmethyl amines, careful inspection of a reaction mixture containing pyrrolidine and the ketone showed the formation of a 2′-hydroxyacetophenone self-condensation product. 

Aware of the limitations of the available methods for the preparation of methanodibenzo[*b*,*f*][1,5]dioxocin, and intrigued by the singular reactivity of pyrrolidine in promoting the self-condensation reaction, we set out to investigate and optimize the reaction conditions to efficiently construct such a polycyclic core from the self-condensation of three 2′-hydroxyacetophenone molecules. Importantly, previously reported attempts to dimerize β-hydroxy ketones using acidic/dehydrating conditions proved unsuccessful, although 6H,12H-6,12-epoxydibenzo[*b*,*f*][1,5]dioxocins could be obtained from the dimerization of salicylaldehydes [43,44].

## 2. Results and Discussion

We began our synthetic efforts to optimize the reaction conditions after obtaining methanodibenzo[*b*,*f*][1,5]dioxocin **1** at a 9% yield in a failed attempt to aminate 2′-hydroxyacetophenone with pyrrolidine under hydrosilylation conditions, followed by crystallization and unambiguous characterization of the product through single crystal X-ray diffraction analysis (Table 1). The crystal structure of **1** showed an intramolecular hydrogen bond and similar geometric features of the methanodibenzo[*b*,*f*][1,5]dioxocin core to 7,9-dimethoxy-6H,12H-6,12-methanodibenzo[*b*,*f*][1,5]dioxocin-4-ol [26], except for the interplanar angle between benzene rings. The interplanar angle was almost ten degrees higher value in **1**, which was rather close to orthogonality (~88°) and corresponded to the value found from (*E*)-2-(4-((12-methyl-6H,12H-6,12-methanodibenzo[*b*,*f*][1,5]dioxocin-6-yl)methylene)-4H-chromen-2-yl)phenol [45].

Using 2-hydroxyacetophenone as substrate, we investigated the reaction conditions in the hydrosilylation protocol and their effect on the formation of compound **1** (Table 1). The initial use of stoichiometric amounts of 2′-hydroxyacetophenone and pyrrolidine in refluxing acetonitrile gave compound **1** with a 24% yield (Table 1, entry 1). The importance of pyrrolidine and molecular sieves (MS) was verified after not detecting the desired product when running the reaction in the absence of any of these components (entries 2 and 3). Replacing pyrrolidine with anhydrous *p*-toluenesulfonic acid, or use of pyrrolidine as a solvent led to similar outcomes (entries 4 and 5). Decreasing the amount of pyrrolidine to 0.3 equiv. had a positive effect on the formation of **1**, with a yield of 42% (entry 6). Harsher reaction conditions, namely using a sealed tube at 80 °C in the absence of solvent and increased reaction times, allowed formation of product with up to 59% yield (entry 7). Decreasing the amount of the pyrrolidine catalyst did not improve the yield of the desired product (Table 1, entries 8–10). Other reaction conditions tested, including use of additives such as acetic acid or Cu(OAc)_2_, replacing molecular sieves beads with powder and using different amounts of sieves, invariably led to lower yields. 

At this point, the best conditions identified were the use of 30 mol% pyrrolidine to catalyze the transformation and a solvent-free protocol. While the model compound tested is liquid at room temperature, many 2′-hydroxyacetophenones are solid, which could likely pose reproducibility, heat and mass transfer issues in a solvent free process. Furthermore, after considerable product formation, it is solidified in the mixture, trapping the molecular sieves and hampering efficient stirring of the reaction mixture. Due to this, we decided to search for solvents suitable for the reaction, which would solubilize the starting materials and prevent decomposition of the product. With the aim of getting both the starting material and product soluble in the reaction media, more polar solvents were initially tested in both vessel types, sealed tube and round bottom flask, under argon (Table 2). Interestingly, ethanol, the only protic solvent tested, failed to provide any of the product, and dichloroethane did not provide more than a 15% yield of **1** after 24 h. Other polar solvents, such as acetonitrile and methyl *tert-*butylether, could provide the product in yields of up to 43%, but slightly better yields could be obtained for hexane, while toluene and dioxane were comparable. From the reaction optimizations so far, it appeared that hexane as a solvent favored the yield of compound **1** compared to the others screened in both closed and reflux reaction conditions. A more dilute reaction media in hexane did not affect the yield of **1** considerably (Table 2, entry 7). Thus, we identified the use of 0.3 equiv. of pyrrolidine as a catalyst in refluxing hexane to be the optimal reaction conditions for the synthesis of **1** based on the optimization reactions carried out and also our quest to avoid reactions under neat conditions. 

**Scheme 3:**
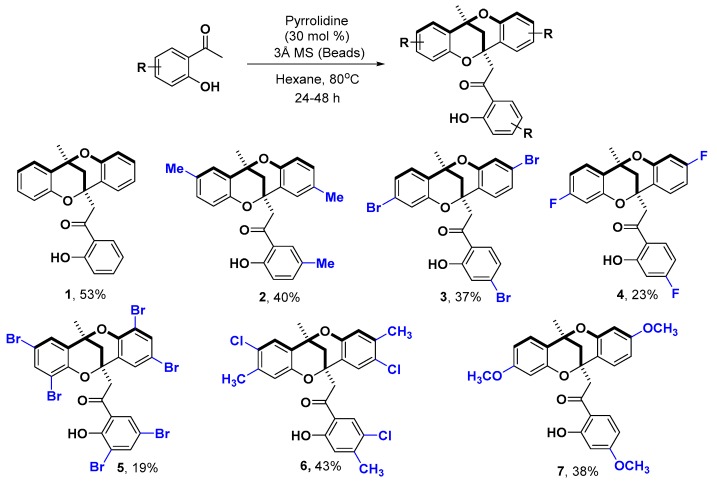
Synthesis of substituted methanodibenzo[*b*,*f*][1,5]dioxocin derivatives.

With the established optimal reaction conditions in hand, we proceeded to expand the substrate scope to other substituted 2′-hydroxyacetophenones (Scheme 3). Notwithstanding the modest yields, we were pleased to observe the formation of methanodibenzo[*b*,*f*][1,5]dioxocin derivatives **2**–**7** without the need to use any metals or strongly acidic conditions. The protocol was demonstrated to be suitable for obtaining alkyl-substituted methanodibenzo[*b*,*f*][1,5]dioxocin **2**, the halide substituted derivatives **3**–**6** or the electron rich methoxy derivative **7**. When attempting to verify the suitability of this method for strongly electron-withdrawing groups, nitro substituted 2′-hydroxyacetophenones were tested, resulting only in the isolation of traces of the reduced aniline and unreacted starting material. Also, all attempts to obtain the methanodibenzo[*b*,*f*][1,5]dioxocin analogue of other 2′-hydroxyketones, such as 2′-hydroxypropiophenone or 2′-hydroxy-3-phenylpropiophenone proved futile, therefore limiting this protocol to pyrrolidine catalyzed self-condensation of 2′-hydroxyacetophenones. 

Although the details of the mechanisms involved require full clarification, the absence of similar dioxocin products when employing other cyclic secondary amines, such as indoline, morpholine and tetrahydroquinoline, using our hydrosilylation protocol, suggests that an enamine is likely to be involved. Pyrrolidine derived enamines are known to be more reactive that other cyclic amines [46,47], and the protocol for reductive amination from 2′-hydroxyacetophenone and this amine starts with iminium formation at room temperature under neat conditions [48]. Moreover, the same amine was previously reported to promote the formation of 4-chromanones from 2′-hydroxyacetophenones and aliphatic aldehydes and ketones [49]. 

## 3. Materials and Methods

### 3.1. General Considerations

All syntheses were carried out in oven-dried glassware under an inert atmosphere. All solvents used were left standing over 3 Å molecular sieves and used without further drying. All other reagents were purchased from Sigma-Aldrich or TCI and used without further purification. Reactions were monitored through thin-layer chromatography (TLC) with commercial silica gel plates (Merck silica gel, 60 F254). Visualization of the developed plates was performed under UV lights at 254 nm and by staining with cerium ammonium molybdate and vanillin. Flash column chromatography was performed on silica gel 60 (40–63 µm) as stationary phase. NMR spectra were recorded with JEOL ECZR 500 instruments using CDCl_3_ as solvent. Chemical shifts (δ) were reported in ppm and referenced to the CDCl_3_ residual peak (δ 7.26) or tetramethylsilane (TMS) peak (δ 0.00) for ^1^H NMR and to CDCl_3_ (δ 77.16) for ^13^C NMR. The following abbreviations were used to describe peak splitting patterns: s = singlet, d = doublet, t = triplet and m = multiplet. Coupling constants, *J*, were reported in hertz (Hz). High-resolution mass spectrometry spectra were recorded on a Waters ESI-TOF MS spectrometer. 

### 3.2. General Procedure for the Synthesis of Substituted Methanodibenzo[b,f][1,5]Dioxocin Derivatives ***1**–**7***

In a 10 mL round bottom flask equipped with a condenser, the corresponding 2′-hydroxyacetophenone (6.22 mmol) was heated in hexane (5 mL) for 5–10 min to dissolve completely, after which pyrrolidine (2.08 mmol, 0.33 equiv) and molecular sieves (3 Å beads, 362 mg) were added while stirring under argon. The resulting mixture was refluxed at 80 °C for 24–48 h and then allowed to cool to room temperature. After cooling to room temperatute (r. t.), ethyl acetate was added to the reaction mixture, followed by saturated NH_4_Cl (15 mL). The aqueous layer was extracted with ethyl acetate (3 × 20 mL) and the combined organic layers were dried over MgSO_4_, filtered out and the solvent was removed under reduced pressure. The residue was then purified by flash column chromatography on silica (Hexane:EtOAc 98:2) to give the desired product. 1H and 13C spectra of all compounds, **1**–**7** is available in the supplementary material.

*1-(2-hydroxyphenyl)-2-(12-methyl-6H,12H-6,12-methanodibenzo[b,f][1,5]dioxocin-6-yl)ethan-1-one* (**1**). Following the general procedure, 2′-hydroxyacetophenone (845 mg, 6.22 mmol) and pyrrolidine (170 µL, 2.08 mmol) in hexane (1.5 mL) refluxed for 24 h. The purified product was obtained as a pale yellow crystalline solid at a 53% yield (406 mg, 1.09 mmol). ^1^H NMR (CDCl_3_, 500 MHz): δ = 12.12 (s, 1H, OH), 8.08 (d, *J* = 7.4 Hz, 1H. ArH), 7.44–7.55 (m, 2H, ArH), 7.40 (d, *J* = 7.4 Hz, 1H, ArH), 7.06–7.16 (m, 2H, ArH), 6.93–7.01 (m, 2H, ArH), 6.78–6.92 (m, 3H, ArH), 6.71 (d, *J* = 8.0 Hz, 1H, ArH), 4.16 (d, *J* = 16.0 Hz, 1H, CH_2_), 3.79 (d, *J* = 16.0 Hz, 1H, CH_2_), 2.68 (d, *J* = 13.7 Hz, 1H, CH_2_), 2.26 (d, *J* = 13.7 Hz, 1H, CH_2_), 1.87 ppm (s, 3H, CH_3_). ^13^C NMR (CDCl_3_, 126 MHz): δ = 202.6, 162.6, 154.0, 152.8, 136.6, 130.7, 129.9, 129.7, 126.5, 125.5, 124.2, 122.7, 120.8, 120.6, 119.9, 118.8, 118.4, 117.4, 116.9, 72.1, 70.3, 45.1, 38.7, 25.2. HRMS (ESI) *m*/*z*: Calculated for C_24_H_21_O_4_ [M + H]^+^ 373.1440. Found 373.1411.

*1-(2-hydroxy-5-methylphenyl)-2-(2,8,12-trimethyl-6H,12H-6,12-methanodibenzo[b,f][1,5]dioxocin-6-yl)ethan-1-one* (**2**). Following the general procedure, 2′-hydroxy-5′-methylacetophenone (934 mg, 6.22 mmol) and pyrrolidine (170 µL, 2.08 mmol) in hexane (5 mL) refluxed for 48 h. The purified product was obtained as a pale yellow crystalline solid at a 40% yield (341 mg, 0.82 mmol). ^1^H NMR (CDCl_3_, 500 MHz): δ = 11.94 (s, 1H, OH), 7.86 (d, *J* = 1.7 Hz, 1H, ArH), 7.31 (dd, *J* = 8.6, 2.3 Hz, 1H, ArH), 7.26 (d, *J* = 1.7 Hz, 1H, ArH), 7.15 (d, *J* = 2.3 Hz, 1H, ArH), 6.89–6.95 (m, 2H, ArH), 6.87 (d, *J* = 8.6 Hz, 1H, ArH), 6.69 (d, *J* = 8.0 Hz, 1H, ArH), 6.60 (d, *J* = 8.0 Hz, 1H, ArH), 4.09 (d, *J* = 16.0 Hz, 1H, CH_2_), 3.80 (d, *J* = 16.0 Hz, 1H, CH_2_), 2.64 (d, *J* = 13.7 Hz, 1H, CH_2_), 2.38 (s, 3H, CH_3_), 2.22 (s, 3H, CH_3_), 2.19 (s, 3H, CH_3_), 2.15 (d, *J* = 13.7 Hz, 1H, CH_2_), 1.83 ppm (s, 3H, CH_3_). ^13^C NMR (CDCl_3_, 126 MHz): δ = 202.8, 160.8, 152.1, 150.9, 137.9, 131.0, 130.8, 130.7, 130.2, 129.9, 128.1, 127.0, 125.9, 124.2, 122.7, 119.9, 118.4, 117.3, 116.8, 72.2, 70.5, 45.6, 39.1, 25.6, 20.9, 20.8, 20.7. HRMS (ESI) *m*/*z*: Calculated for C_27_H_27_O_4_ [M + H]^+^ 415.1909. Found 415.1850. 

*1-(4-bromo-2-hydroxyphenyl)-2-(3,9-dibromo-12-methyl-6H,12H-6,12-methanodibenzo[b,f][1,5]dioxocin-6-yl)ethan-1-one* (**3**). Following the general procedure, 4′-bromo-2′-hydroxyacetophenone (192 mg, 0.90 mmol) and pyrrolidine (24 µL, 0.3 mmol) in hexane (1 mL) refluxed for 48 h. The purified product was obtained as a white crystalline solid at a 37% yield (68 mg, 0.11 mmol). ^1^H NMR (CDCl_3_, 500 MHz): δ = 12.09 (s, 1H, OH), 7.86 (d, *J* = 8.6 Hz, 1H, ArH), 7.32 (d, *J* = 8.6 Hz, 1H, ArH), 7.17–7.22 (m, 2H, ArH), 7.13 (dd, *J* = 8.6, 1.7 Hz, 1H, ArH), 7.03 (dd, *J* = 8.3, 2.0 Hz, 1H, ArH), 6.96–7.00 (m, 2H, ArH), 6.86 (d, *J* = 1.7 Hz, 1H, ArH), 4.06 (d, *J* = 16.0 Hz, 1H, CH_2_), 3.69 (d, *J* = 16.0 Hz, 1H, CH_2_), 2.64 (d, *J* = 13.7 Hz, 1H, CH_2_), 2.20 (d, *J* = 14.3 Hz, 1H, CH_2_), 1.82 ppm (s, 3H, CH_3_). ^13^C NMR (CDCl3, 126 MHz): δ = 201.7, 163.3, 155.1, 153.7, 131.7, 131.6, 128.2, 127.0, 124.7, 124.4, 123.7, 123.4, 123.3, 122.8, 122.0, 121.6, 121.0, 120.3, 118.9, 72.6, 70.9, 45.1, 38.5, 25.2. HRMS (ESI) *m*/*z*: Calculated for C_24_H_16_Br_3_O_4_ [M − H]^−^ 606.8580, 608.8561. Found 606.8564, 608.8533. 

*2-(3,9-difluoro-12-methyl-6H,12H-6,12-methanodibenzo[b,f][1,5]dioxocin-6-yl)-1-(4-fluoro-2-hydroxyphenyl)ethan-1-one* (**4**). Following the general procedure, 4′-fluoro-2′-hydroxyacetophenone (959 mg, 6.22 mmol) and pyrrolidine (170 µL, 2.08 mmol) in hexane (3 mL) refluxed for 48 h. The purified product was obtained as a yellow crystalline solid at a 23% yield (199 mg, 0.47 mmol). ^1^H NMR (CDCl_3_, 500 MHz): δ = 12.39 (d, *J* = 1.7 Hz, 1H, OH), 8.06 (dd, *J* = 9.2, 6.3 Hz, 1H, ArH), 7.44 (dd, *J* = 8.6, 6.3 Hz, 1H, ArH), 7.30–7.39 (m, 1H, ArH), 6.56–6.74 (m, 4H, ArH), 6.51 (dd, *J* = 9.7, 2.9 Hz, 1H, ArH), 6.41 (dd, *J* = 10.0, 2.6 Hz, 1H, ArH), 4.09 (d, *J* = 16.0 Hz, 1H, CH_2_), 3.69 (d, *J* = 16.0 Hz, 1H, CH_2_), 2.65 (d, *J* = 13.7 Hz, 1H, CH_2_), 2.23 (d, *J* = 13.7 Hz, 1H, CH_2_), 1.85 ppm (s, 3H, CH_3_). ^13^C NMR (CDCl_3_, 126 MHz): δ = 201.2, 167.8 (d, ^1^*J_C-F_* = 258.3 Hz), 165.6 (d, ^3^*J_C-F_* = 13.9 Hz), 163.6 (d, ^1^*J_C-F_* = 249.5 Hz), 163.4 (d, ^1^*J_C-F_* = 246.9 Hz), 155.7 (d, ^3^*J_C-F_* = 12.6 Hz), 154.3 (d, ^3^*J_C-F_* = 12.6 Hz), 133.4 (d, ^3^*J_C-F_* = 12.6 Hz), 128.3 (d, ^3^*J_C-F_* = 10.1 Hz), 127.2 (d, ^3^*J_C-F_* = 10.1 Hz), 120.4 (d, ^4^*J_C-F_* = 3.8 Hz), 118.8 (d, ^4^*J_C-F_* = 3.8 Hz), 117.3 (d, ^4^*J_C-F_* = 2.5 Hz), 108.9 (d, ^2^*J_C-F_* = 21.4 Hz), 108.6 (d, ^2^*J_C-F_* = 21.4 Hz), 107.6 (d, ^2^*J_C-F_* = 23.9 Hz) 105.3 (d, ^2^*J_C-F_* = 23.9 Hz), 104.7 (d, ^2^*J_C-F_* = 23.9 Hz), 104.1 (d, ^2^*J_C-F_* = 23.9 Hz), 72.7, 71.0, 45.3, 38.7, 25.4. HRMS (ESI) *m*/*z*: Calculated for C_24_H_16_F_3_O_4_ [M − H]^−^ 425.1001. Found 425.0961. 

*1-(3,5-dibromo-2-hydroxyphenyl)-2-(2,4,8,10-tetrabromo-12-methyl-6H,12H-6,12-methanodibenzo[b,f][1,5]dioxocin-6-yl)ethan-1-one* (**5**). Following the general procedure, 3′,5′-dibromo-2′-hydroxyacetophenone (1.83 g, 6.22 mmol) and pyrrolidine (170 µL, 2.08 mmol) in hexane (5 mL) refluxed for 48 h. The purified product was obtained as a green crystalline solid at a 19% yield (332 mg, 0.39 mmol). ^1^H NMR (CDCl_3_, 500 MHz): δ = 12.67 (s, 1H, OH), 8.29 (d, *J* = 2.3 Hz, 1H, ArH), 7.92 (d, *J* = 2.9 Hz, 1H, ArH), 7.59 (d, *J* = 2.3 Hz, 1H, ArH), 7.55 (d, *J* = 2.3 Hz, 1H, ArH), 7.51 (dd, *J* = 4.0, 2.3 Hz, 2H, ArH), 4.38 (d, *J* = 14.9 Hz, 1H, CH_2_), 3.37 (d, *J* = 15.5 Hz, 1H, CH_2_), 2.57 (d, *J* = 14.3 Hz, 1H, CH_2_), 2.18 (d, *J* = 14.3 Hz, 1H, CH_2_), 1.90 (s, 3H, CH_3_). ^13^C NMR (CDCl_3_, 126 MHz): δ = 201.5, 158.7, 150.3, 149.0, 142.5, 136.9, 136.4, 133.5, 129.0, 127.8, 126.8, 125.1, 121.5, 114.1, 113.5, 113.4, 113.1, 112.6, 111.1, 73.4, 71.9, 45.5, 37.8, 25.3. HRMS (ESI) *m*/*z*: Calculated for C_24_H_13_Br_6_O_4_ [M − H]^−^ 842.5875, 844.5855, 846.5837, 848.5820. Found 842.6132, 844.5813, 846.5826 and 848.5784. 

*1-(5-chloro-2-hydroxy-4-methylphenyl)-2-(2,8-dichloro-3,9,12-trimethyl-6H,12H-6,12-methanodibenzo[b,f][1,5]dioxocin-6-yl)ethan-1-one* (**6**). Following the general procedure, 5′-chloro-2′-hydroxy-4′-methylacetophenone (1.15 g, 6.22 mmol) and pyrrolidine (170 µL, 2.08 mmol) in hexane (5 mL) refluxed for 48 h. The purified product was obtained as a brown crystalline solid at a 43% yield (464 mg, 0.90 mmol). ^1^H NMR (CDCl_3_, 500 MHz): δ = 11.90 (s, 1H, OH), 8.00 (s, 1H, ArH), 7.39 (s, 1H, ArH), 7.24–7.31 (m, 1H, ArH), 6.86 (s, 1H, ArH), 6.67 (s, 1H, ArH), 6.60 (s, 1H, ArH), 4.06 (d, *J* = 16.0 Hz, 1H, CH_2_), 3.59 (d, *J* = 16.0 Hz, 1H, CH_2_), 2.55 (d, *J* = 13.7 Hz, 1H, CH_2_), 2.38 (s, 3H, CH_3_), 2.22 ((s, 3H, CH_3_), 2.21 ((s, 3H, CH_3_), 2.13 (d, *J* = 13.7 Hz, 1H, CH_2_), 1.80 ppm (s, 3H, CH_3_). ^13^C NMR (CDCl_3_, 126 MHz): δ = 201.1, 161.3, 152.6, 151.3, 146.5, 138.5, 138.3, 130.6, 126.8, 126.4, 125.8, 124.5, 123.3, 121.8, 120.7, 119.9, 119.3, 119.0, 72.1, 70.6, 45.4, 38.4, 25.4, 21.0, 20.1, 20.0. HRMS (ESI) *m*/*z*: Calculated for C_27_H_22_Cl_3_O_4_ [M − H]^−^ 515.0583, 517.0559. Found 515.0589, 517.0613.

*2-(3,8-dimethoxy-12-methyl-6H,12H-6,12-methanodibenzo[b,f][1,5]dioxocin-6-yl)-1-(2-hydroxy-4-methoxyphenyl)ethan-1-one* (**7**). Following the general procedure, 2′-hydroxy-4′-methoxyacetophenone (1.03 g, 6.22 mmol) and pyrrolidine (170 µL, 2.08 mmol) in hexane (5 mL) refluxed for 48 h. The purified product was obtained as a yellow crystalline solid at a 38% yield (365 mg, 0.79 mmol). ^1^H NMR (CDCl_3_, 500 MHz): δ = 12.69 (s, 1H, OH), 7.96 (d, *J* = 9.2 Hz, 1H, ArH), 7.36 (d, *J* = 9.2 Hz, 1H, ArH), 7.29 (d, *J* = 9.2 Hz, 1H, ArH), 6.49–6.53 (m, *J* = 2.3 Hz, 1H, ArH),) 6.41–6.47 (m, 2H, ArH), 6.40 (d, *J* = 2.9 Hz, 1H, ArH), 6.30 (d, *J* = 2.9 Hz, 1H, ArH), 6.21 (d, *J* = 2.3 Hz, 1H, ArH), 4.06 (d, *J* = 15.5 Hz, 1H, CH_2_), 3.84 (s, 3H, OCH_3_), 3.69 (s, 3H, OCH_3_), 3.68 (s, 3H, OCH_3_), 3.57 (d, *J* = 15.5 Hz, 1H, CH_2_), 2.58 (d, *J* = 13.7 Hz, 1H, CH_2_), 2.20 (d, *J* = 13.2 Hz, 1H, CH_2_), 1.82 ppm (s, 3H, CH_3_). ^13^C NMR (CDCl_3_, 126 MHz): δ = 200.9, 166.4, 165.9, 161.0, 160.9, 155.6, 154.4, 132.8, 127.6, 126.7, 117.0, 115.6, 114.6, 108.5, 108.2, 107.9, 101.4, 101.3, 100.9, 72.7, 71.0, 55.7, 55.3, 55.2, 45.1, 39.2, 25.6. Calculated for C_27_H_25_O_7_ [M − H]^−^ 461.1600 Found 461.1604.

### 3.3. Single Crystal X-Ray Diffraction

The crystal data for **1** were collected on an Agilent SuperNova single-source diffractometer equipped with an Eos CCD detector at 120(2) K using mirror-monochromated Mo-Kα (*λ* = 0.71073 Å) radiation. Data collection (ω scans) and reduction was performed using the program CrysAlisPro [50]. The analytical face-indexing-based absorption correction method was applied. The structure was solved by intrinsic phasing methods [51] and refined by full-matrix least squares on *F*^2^ using SHELXL-2018/1 [52]. Anisotropic displacement parameters were assigned to non-H atoms. All hydrogen atoms (except O–H) were constrained to their idealized positions and refined using riding models with *U*_eq_(H) of 1.5*U*_eq_(C) for terminal methyl groups and of 1.2*U*_eq_(C) for other groups. Hydrogen atoms bonded to O atoms were found from the electron density maps, restrained to their ideal distance (0.84 Å) from the parent atom and refined with *U*_eq_(H) of 1.5*U*_eq_(O). Deposition Number CCDC-1922829 contains the supplementary crystallographic data for this paper. These data are provided free of charge by the joint Cambridge Crystallographic Data Centre and Fachinformationszentrum Karlsruhe Access Structures service www.ccdc.cam.ac.uk/structures.

Crystal data for **1**: C_24_H_20_O_4_ (*M* = 372.40 g/mol), triclinic, space group *P*-1 (no. 2), *a* = 6.8635(3) Å, *b* = 8.5812(4) Å, *c* = 16.4282(7) Å, α = 84.240(4)°, β = 82.066(4)°, γ = 72.435(4)°, *V* = 911.86(7) Å^3^, *Z* = 2, *ρ*_calc_ = 1.356 g/cm^3^, 14,061 reflections measured (3.4° ≤ θ ≤ 29.9°), 4703 unique (*R*_int_ = 0.0329, *R*_sigma_ = 0.0351, *I* > 2*σ*(*I*) = 3781) which were used in all calculations. The final *R*1 was 0.0467 (*I* > 2*σ*(*I*)) and w*R*2 was 0.1222 (all data).

## 4. Conclusions

We have developed a simple, metal-free synthetic route to the highly complex methanodibenzo[*b*,*f*][1,5]dioxocin skeleton from the self-condensation of readily available 2′-hydroxyacetophenones catalyzed by pyrrolidine. Notwithstanding the moderate yields and narrow scope of the transformation, this strategy furnishes, in one-pot, unprecedented 6,12-disubstituted methanodibenzo[*b*,*f*][1,5]dioxocin derivatives with potential usefulness in medicinal chemistry or in the development of bioactive substances.

## Figures and Tables

**Table 1:**
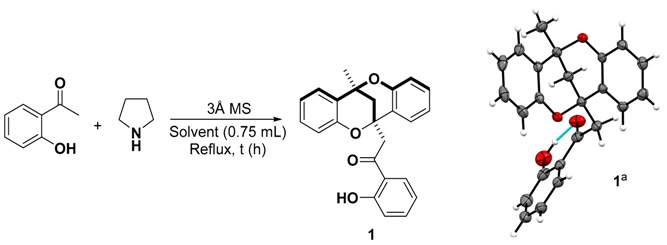
Initial optimization of reaction conditions.

Entry ^b^	Pyrrolidine (equiv.)	Solvent	Conditions	Time (h)	Yield (%) ^c^
1	1	CH_3_CN	Reflux ^d^	18	24
2	1	CH_3_CN	Reflux ^d^, no MS	18	n.d. ^e^
3	none	CH_3_CN	Reflux ^d^	18	n.d. ^e^
4	none	CH_3_CN	dry *p*-TSA (0.2 equiv) ^d^	48	n.d. ^e^
5	-	Pyrrolidine ^g^	Reflux	21	n.d. ^e^
6	0.3	CH_3_CN	Reflux	24	42
7	0.3	none	Sealed tube	24	41 (59) ^f^
8	0.15	none	Sealed tube	24	29
9	0.15	CH_3_CN	Sealed tube	24	36
10	0.05	CH_3_CN	Sealed tube	24	25

^a^ Thermal ellipsoid diagram of the molecular structure of 1-(2-hydroxyphenyl)-2-(12-methyl-6*H*,12*H*-6,12-methanodibenzo[*b*,*f*][1,5]dioxocin-6-yl)ethan-1-one (**1**). ^b^ Unless otherwise noted, 2′-hydroxyacetophenone (6.22 mmol) in refluxing CH_3_CN (0.75 mL), 3 Å molecular sieve (MS, 362 mg) ^c^ Isolated yield. ^d^ 1 mL of solvent used. ^e^ not detected ^f^ after 72 h. ^g^ Pyrrolidine used as solvent (2 mL).

**Table 2:**
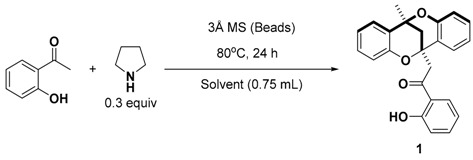
Solvent screening.

Entry	Solvent	Isolated Yield (%)	
		Sealed Tube	Open Vessel
1	CH_3_CN	29	42
2	EtOH	n.d. ^a^	-
3	DMSO	28	-
4	MTBE	32	43
5	1,4-Dioxane	-	30
6	DCE	trace	15
7	Hexane	44 ^b^ (39) ^c^	53 (50) ^d^
8	Toluene	31 ^e^	27
9	Heptane	-	45

^a^ not detected, 2 mL of EtOH used. ^b^ same yield obtained after 48 h. ^c^ Using 1.5 mL of hexane. ^d^ Using 5 mL of hexane and 48 h. ^e^ Powder 3 Å MS and 1.5 mL of toluene were used.

## Supplementary Materials

The following are available online. ^1^H and ^13^C spectra of compounds **1**–**7**.
