# Supplementary material for: Synthesis of 6,12-Disubstituted Methanodibenzo[b,f][1,5]dioxocins: Pyrrolidine Catalyzed Self-Condensation of 2′-Hydroxyacetophenones
Source: Molecules. 2019 Jun 29;24(13):2405. doi: 10.3390/molecules24132405 (PMC6651863; doi:10.3390/molecules24132405)

# (Supplementary Materials)

# Synthesis of 6,12-Disubstituted Methanodibenzo [*b,f*][1,5]dioxocins: Pyrrolidine Catalyzed Self-condensation of 2′-Hydroxyacetophenone

**Benedicta Assoah[1,*], Vesa Riihonen[1], João R. Vale[1,2], Arto Valkonen[3], and Nuno R. Candeias[1,*]**

$^{1}H$ NMR of compound **1**

$CDCl_3$, 500 MHz

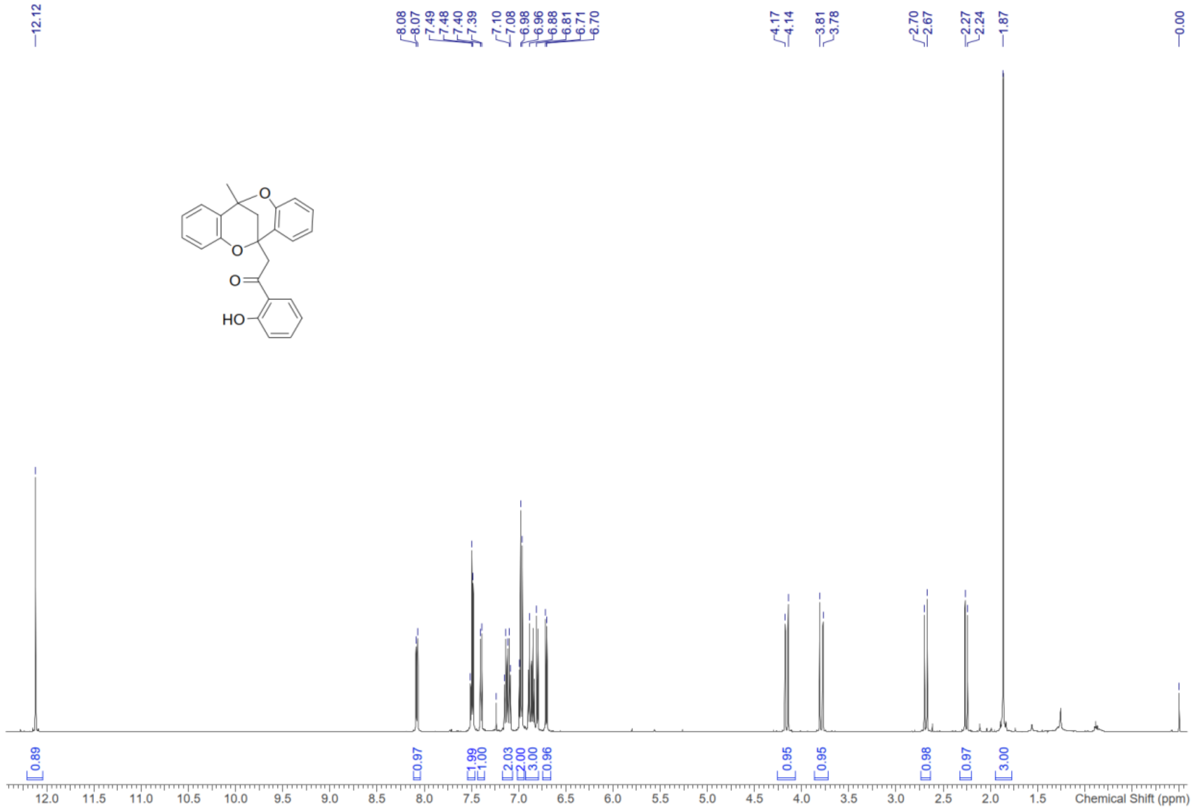


$^{13}C$ NMR of compound **1**

$CDCl_3$, 126 MHz

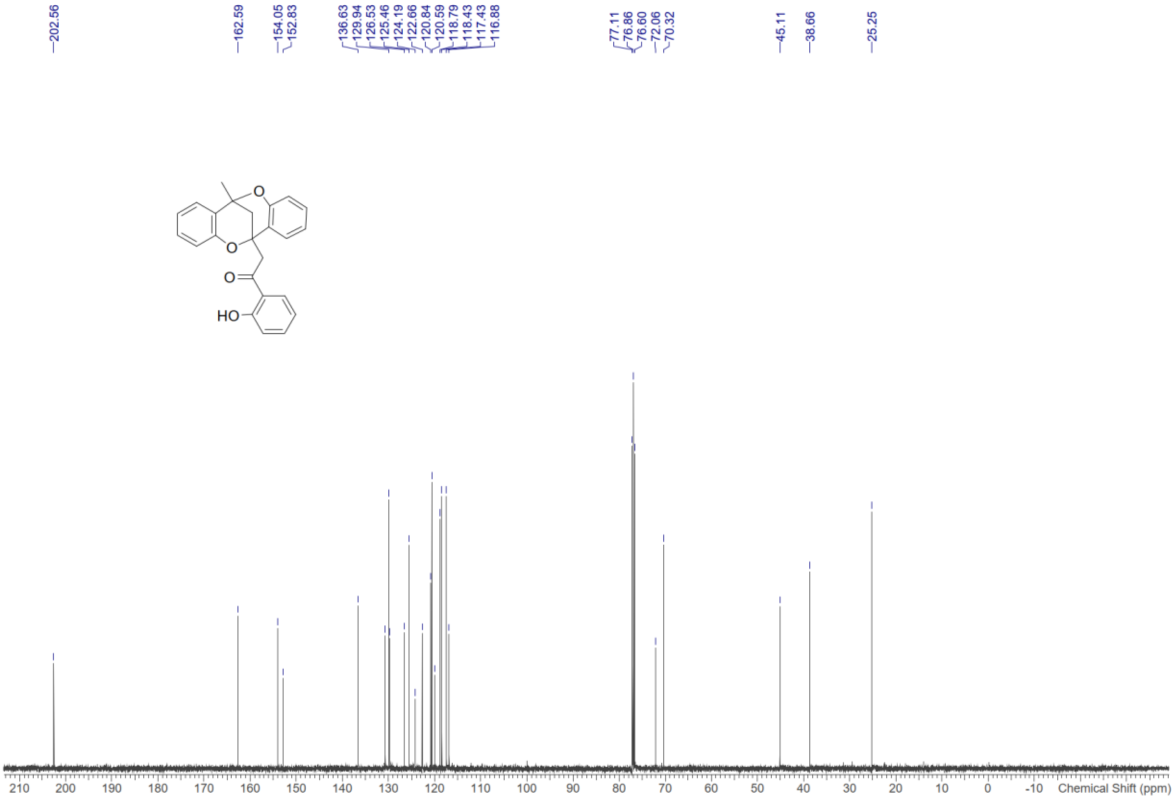

$^{1}H$ NMR of compound **2**

$CDCl_3$, 500 MHz

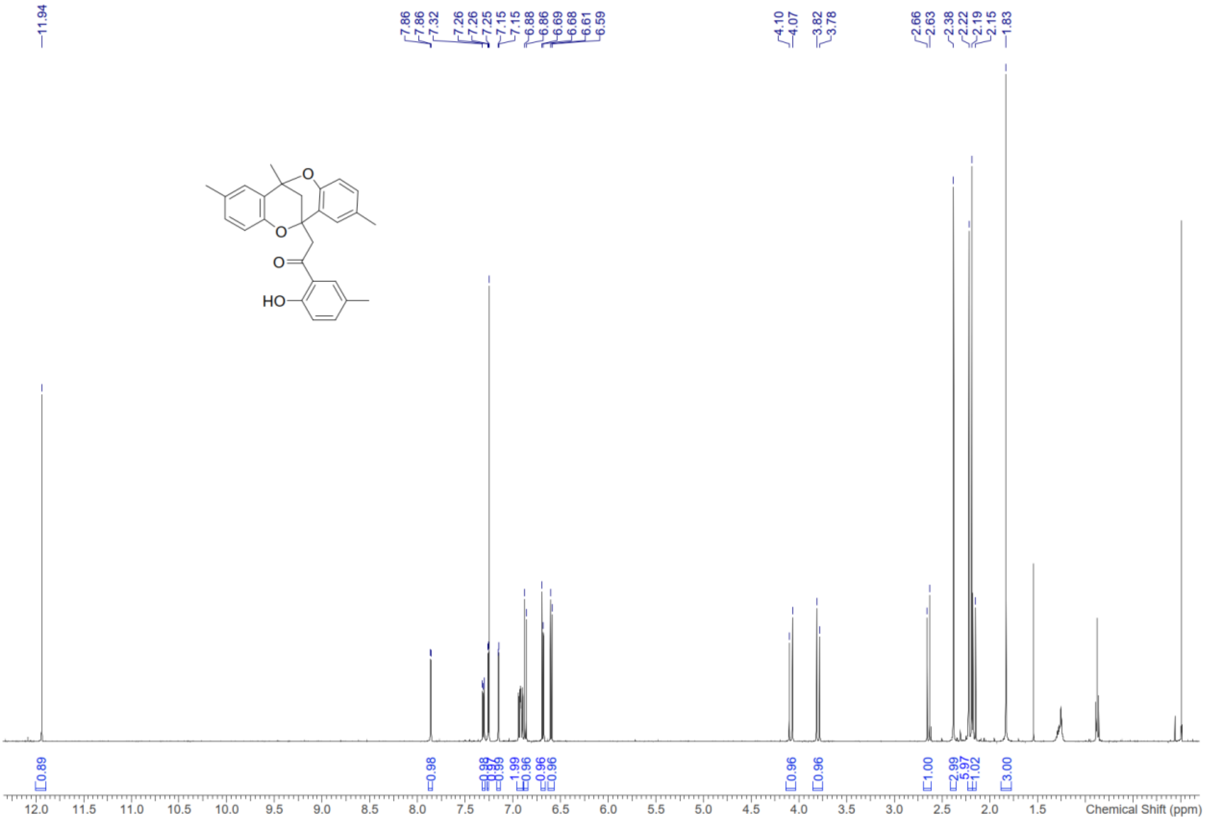

$^{13}C$ NMR of compound **2**

$CDCl_3$, 126 MHz

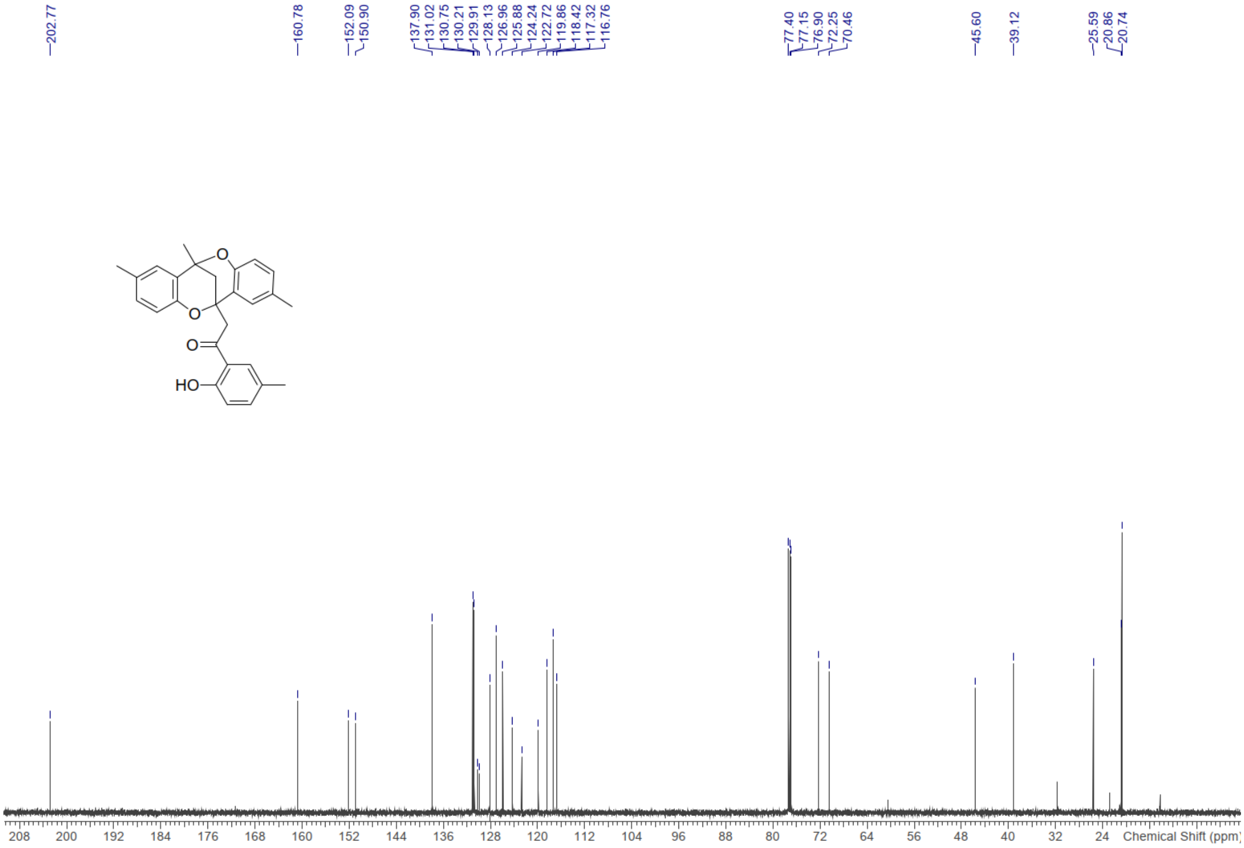

$^{1}$H NMR of compound **3**

$CDCl_3$, 500 MHz

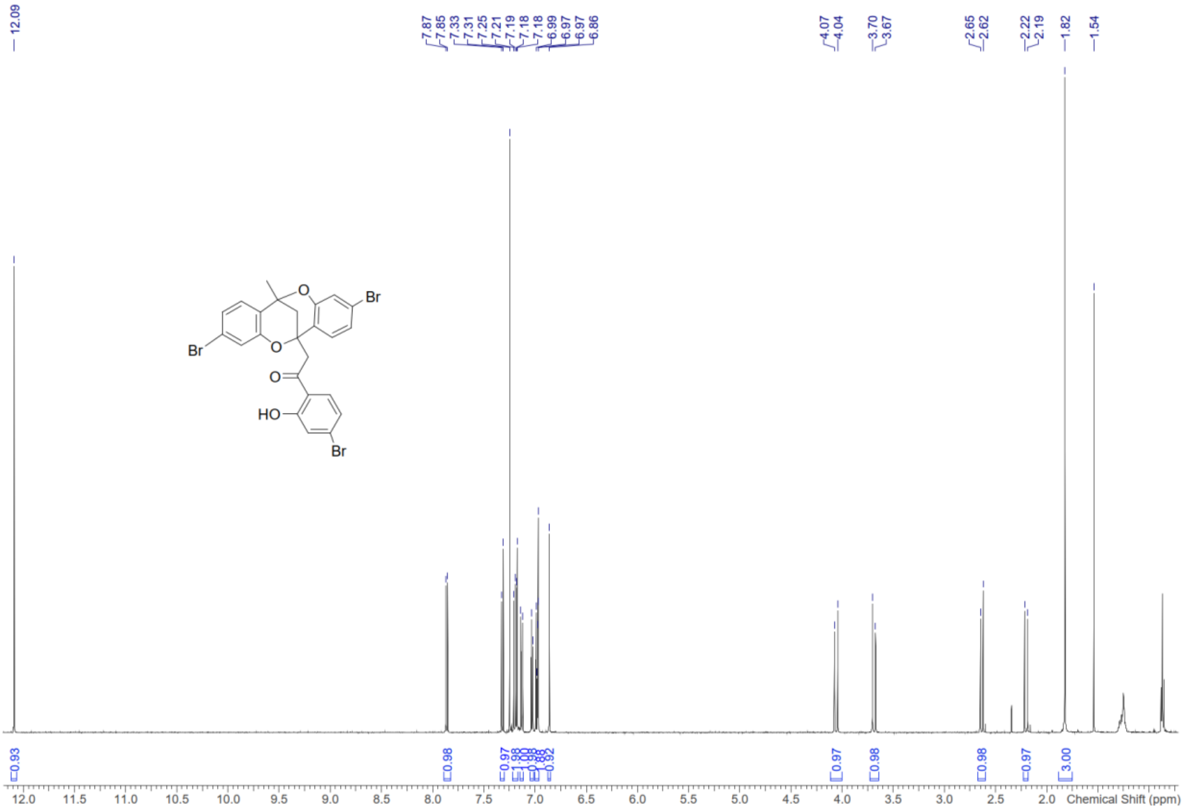


$^{13}$C NMR of compound **3**

$CDCl_3$, 126 MHz

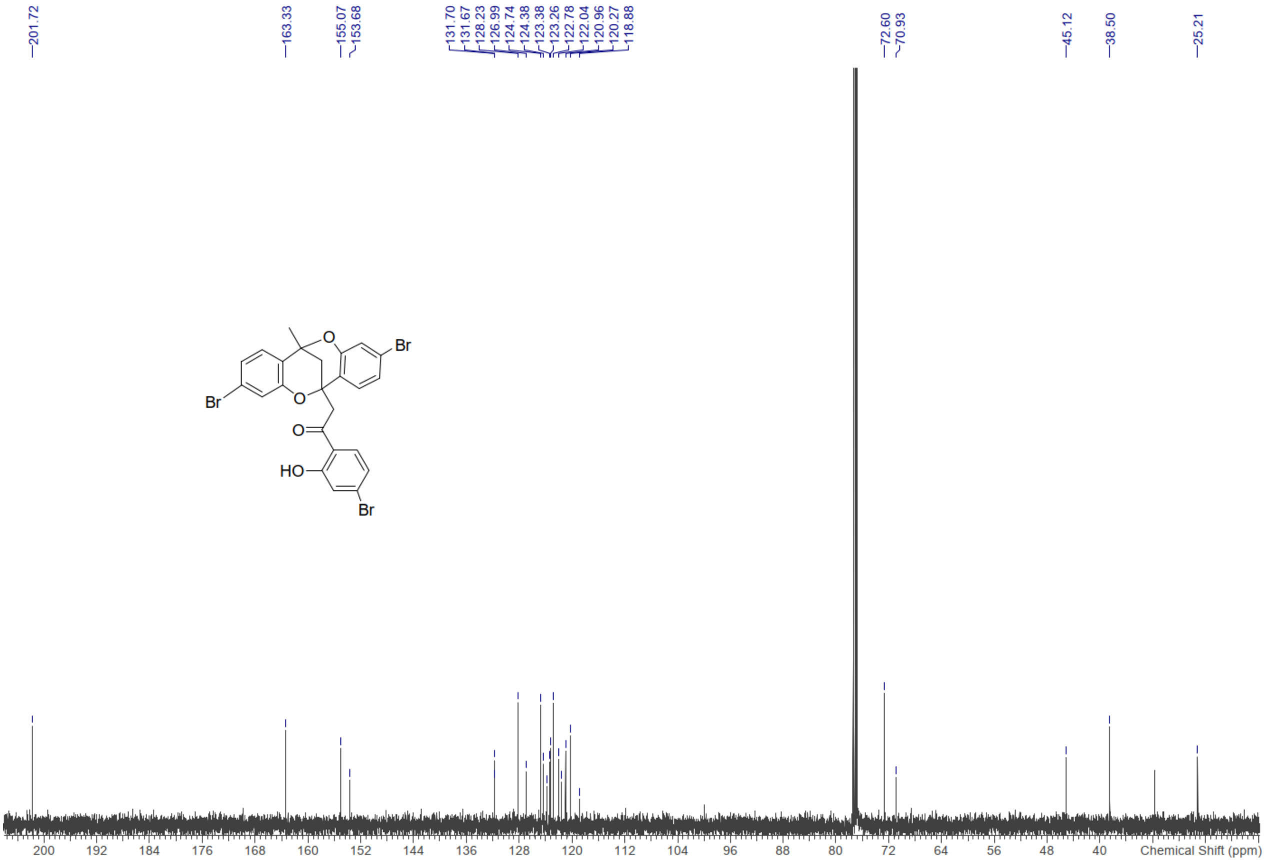

$^{1}$H NMR of compound **4**

$CDCl_3$, 500 MHz

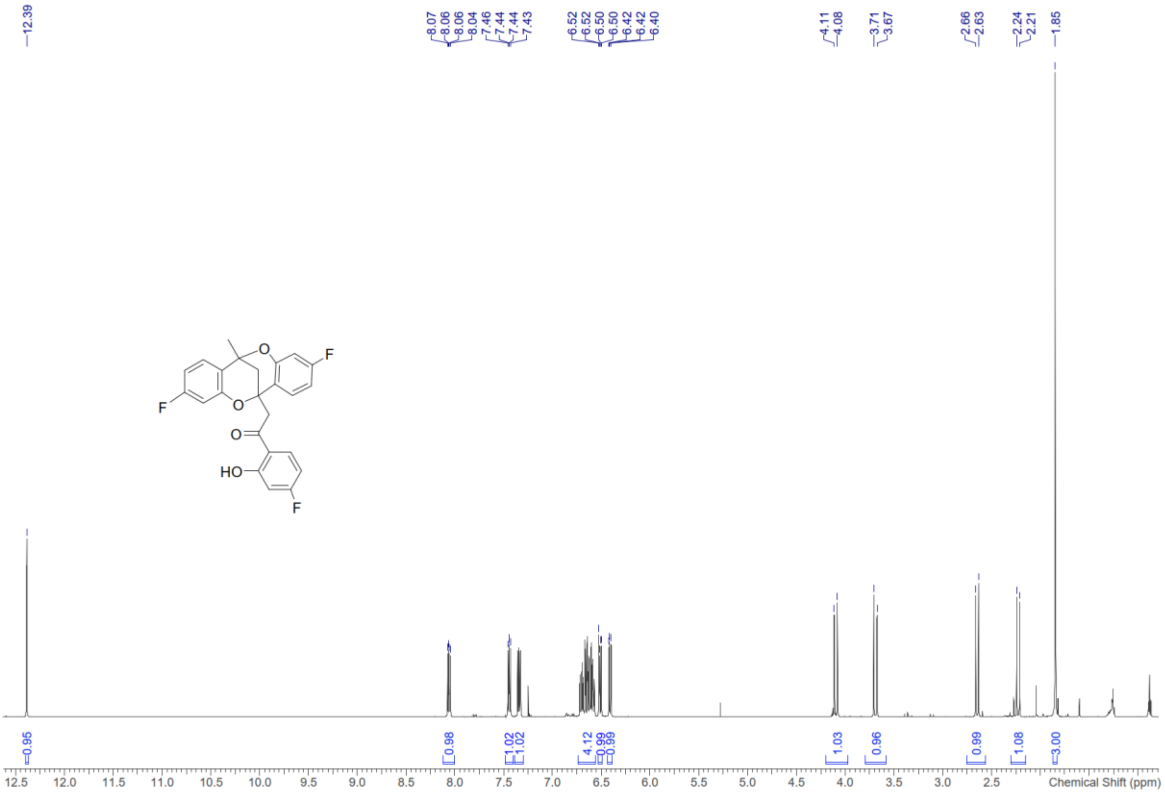


$^{13}$C NMR of compound **4**

$CDCl_3$, 126 MHz

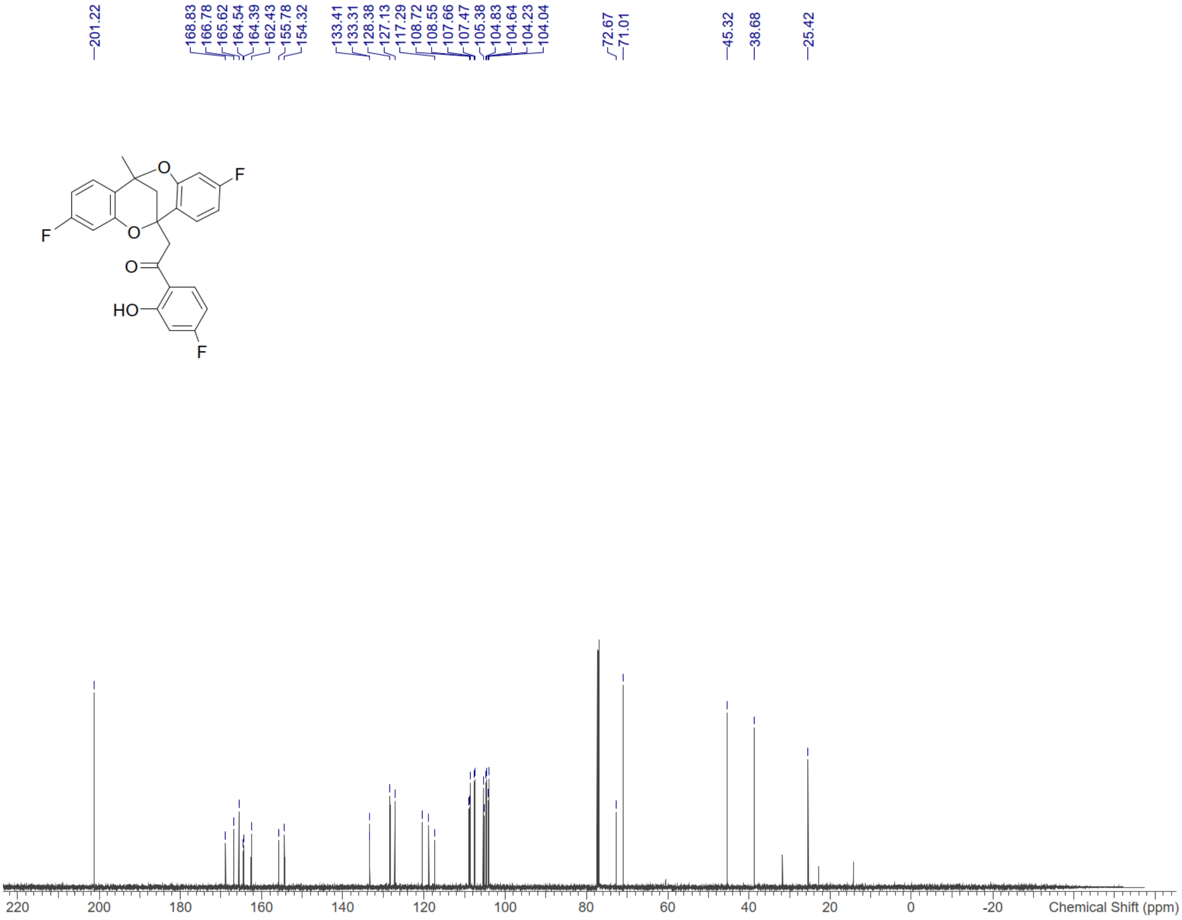

# $^{1}$H NMR of compound **5**

CDCl$_3$, 500 MHz

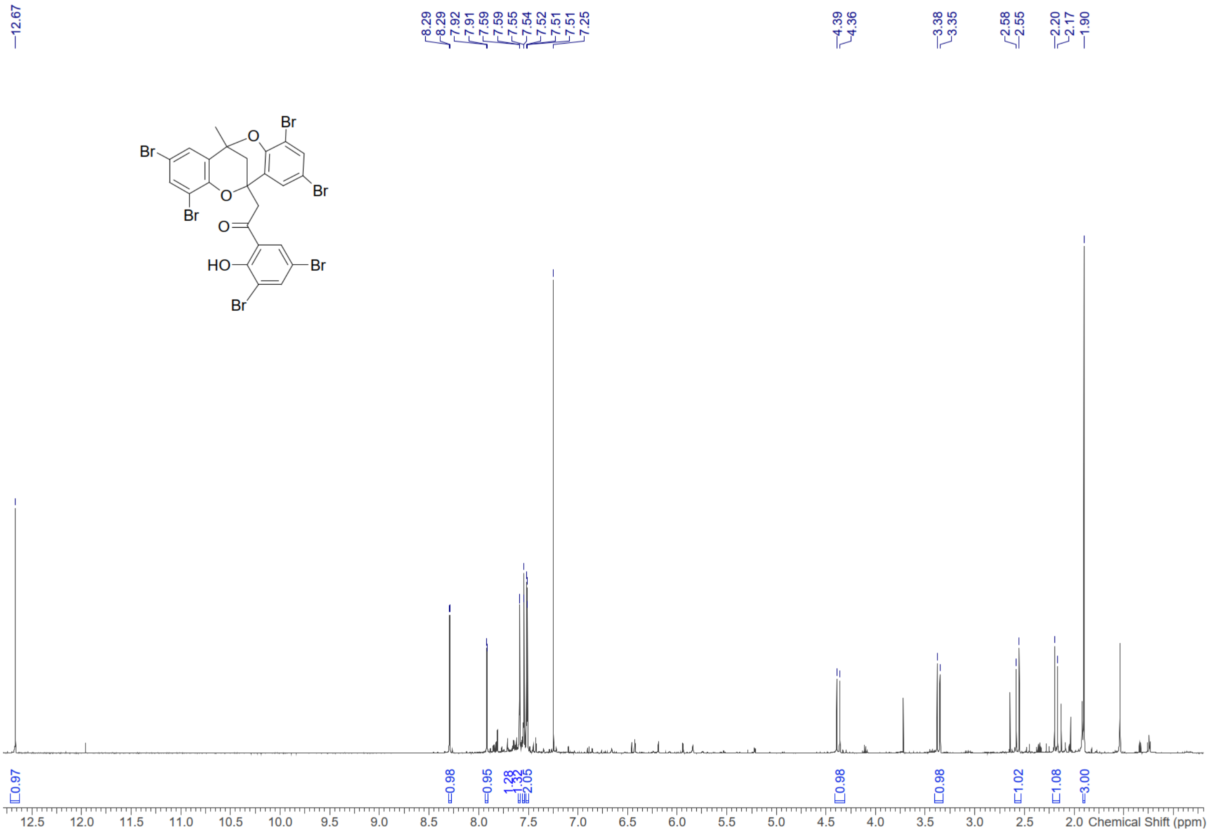


# $^{13}$C NMR of compound **5**

CDCl$_3$, 126 MHz

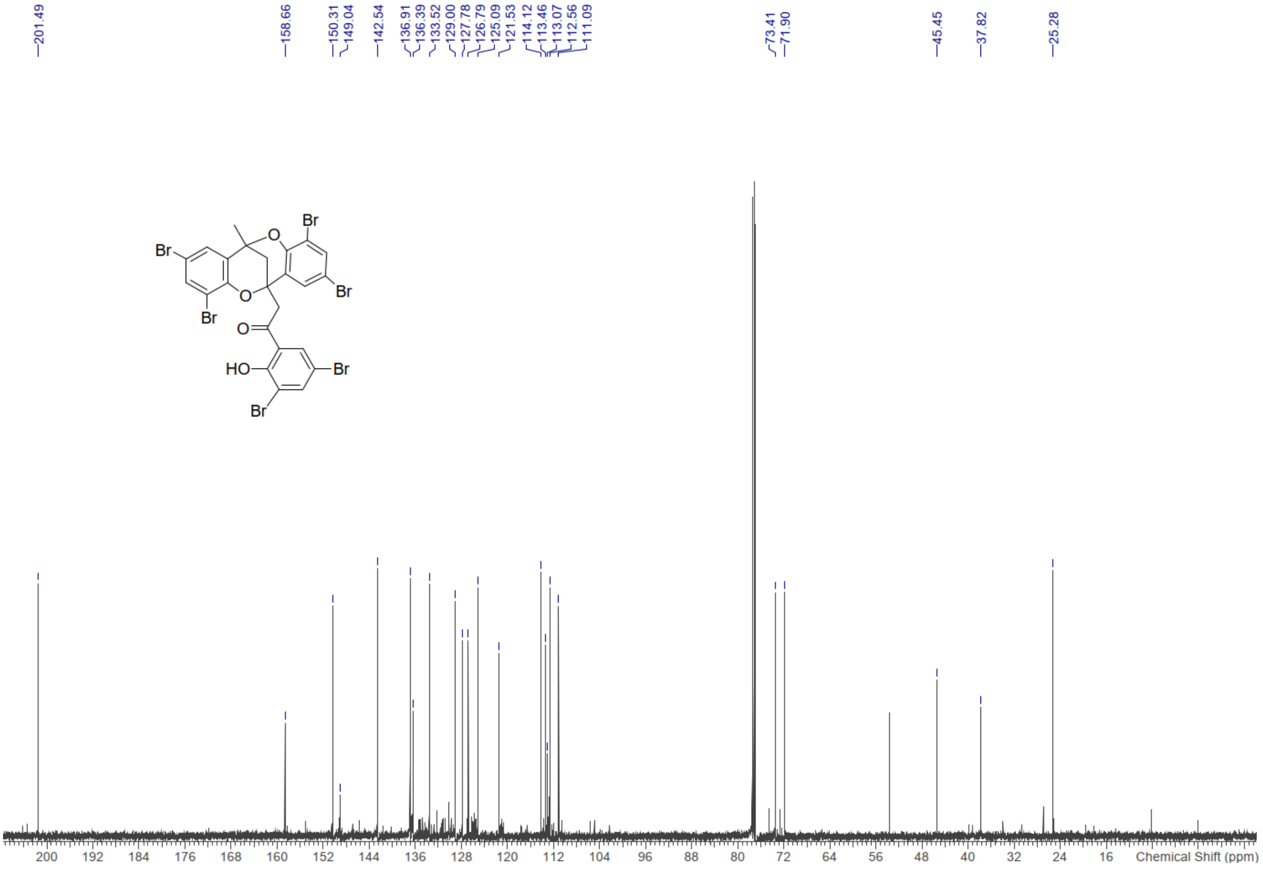

$^{1}$H NMR of compound **6**

$CDCl_3$, 500 MHz

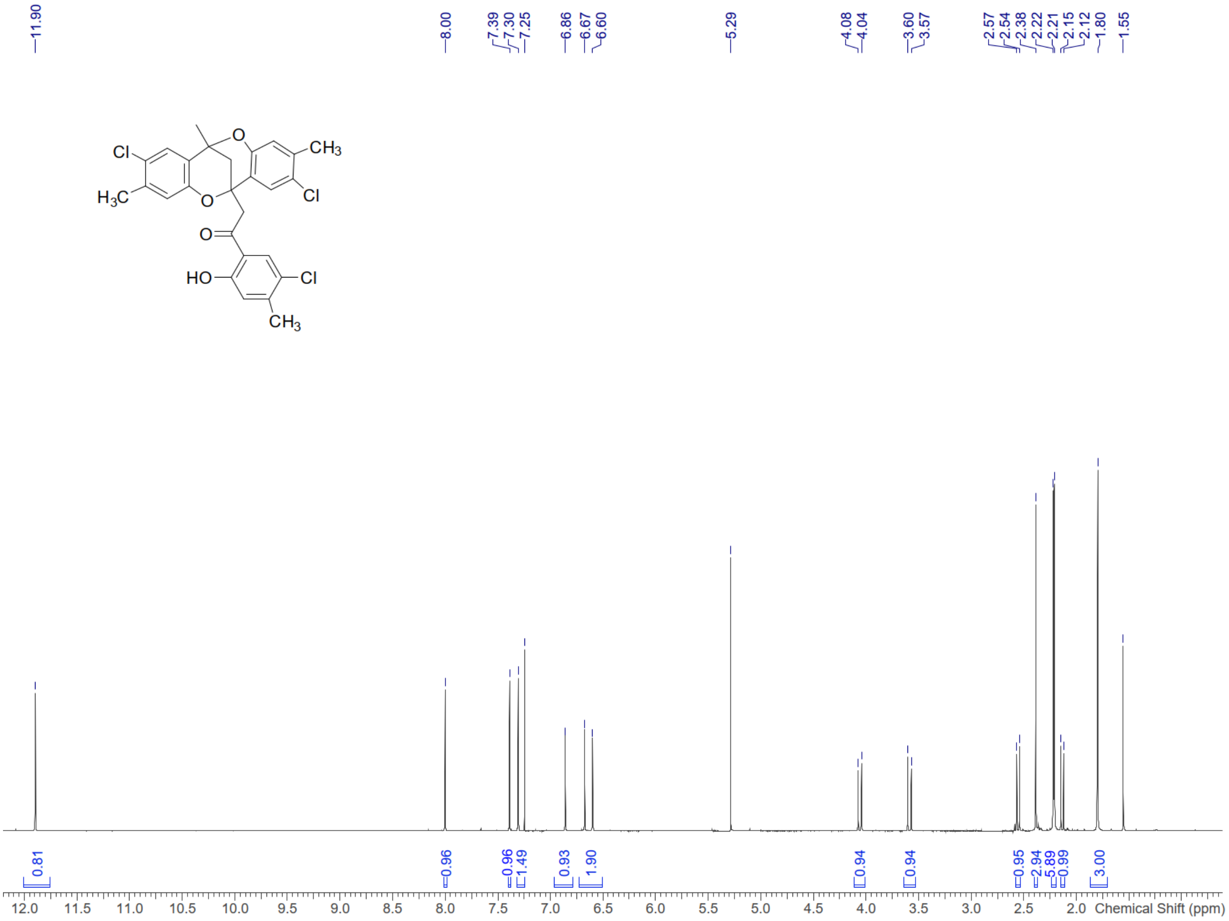

$^{13}$C NMR of compound **6**

$CDCl_3$, 126 MHz

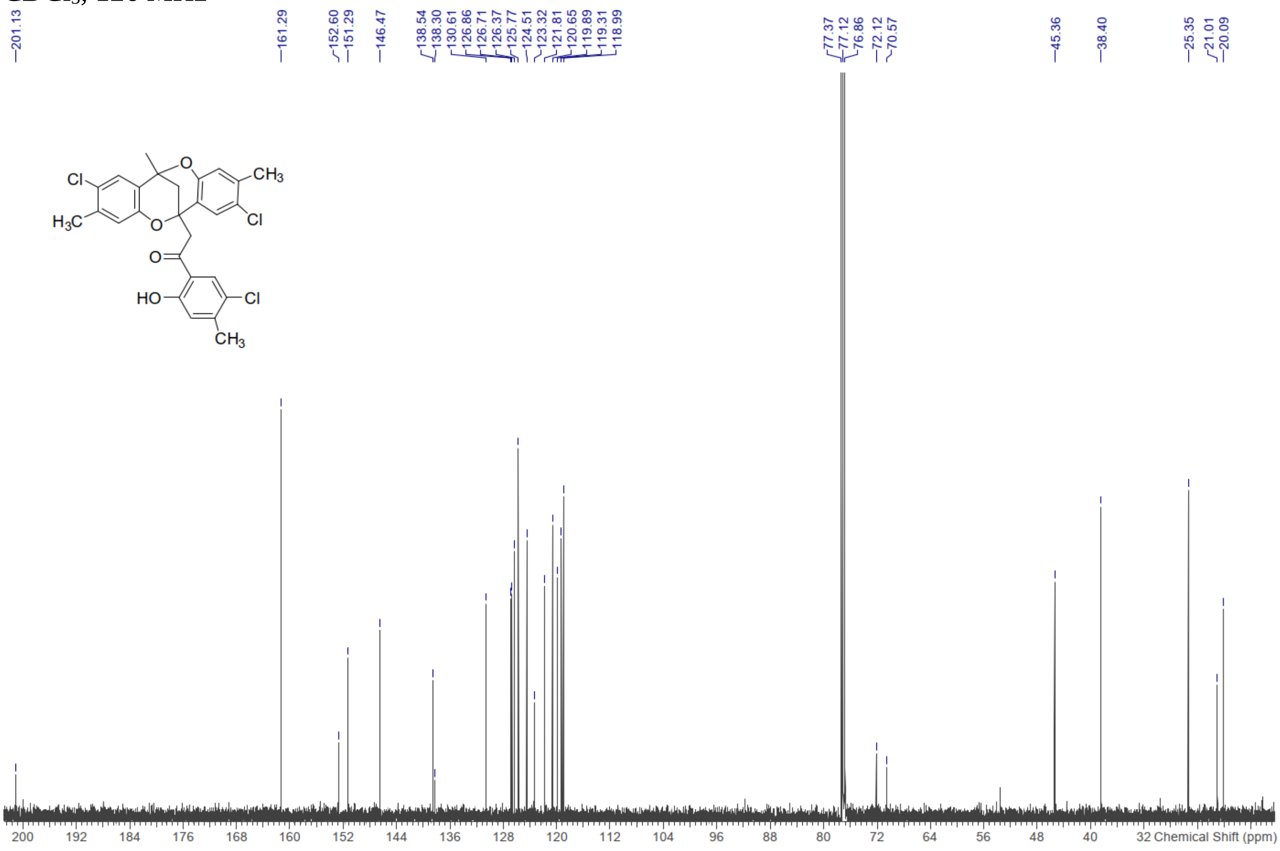

## $^{1}$H NMR of compound **7**

$CDCl_3$, 500 MHz

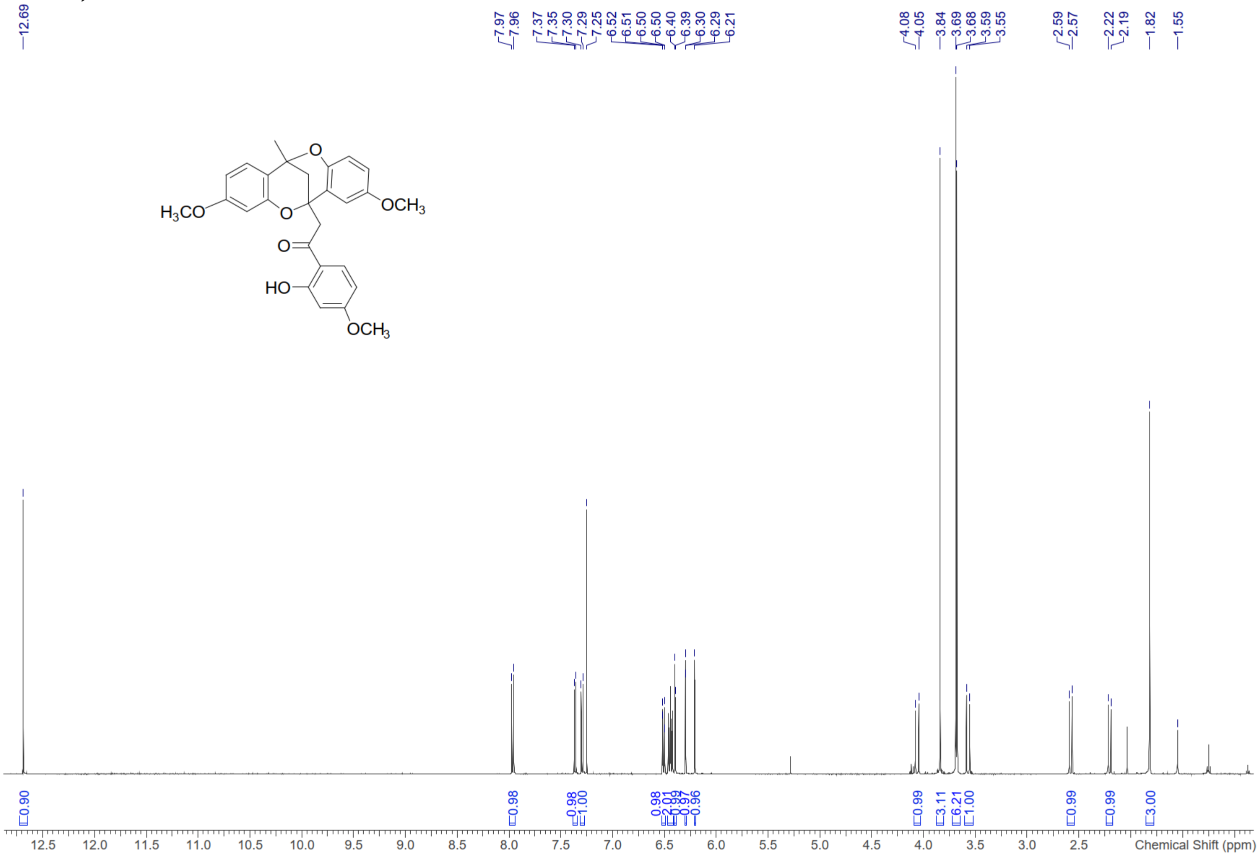


## $^{13}$C NMR of compound **7**

$CDCl_3$, 126 MHz

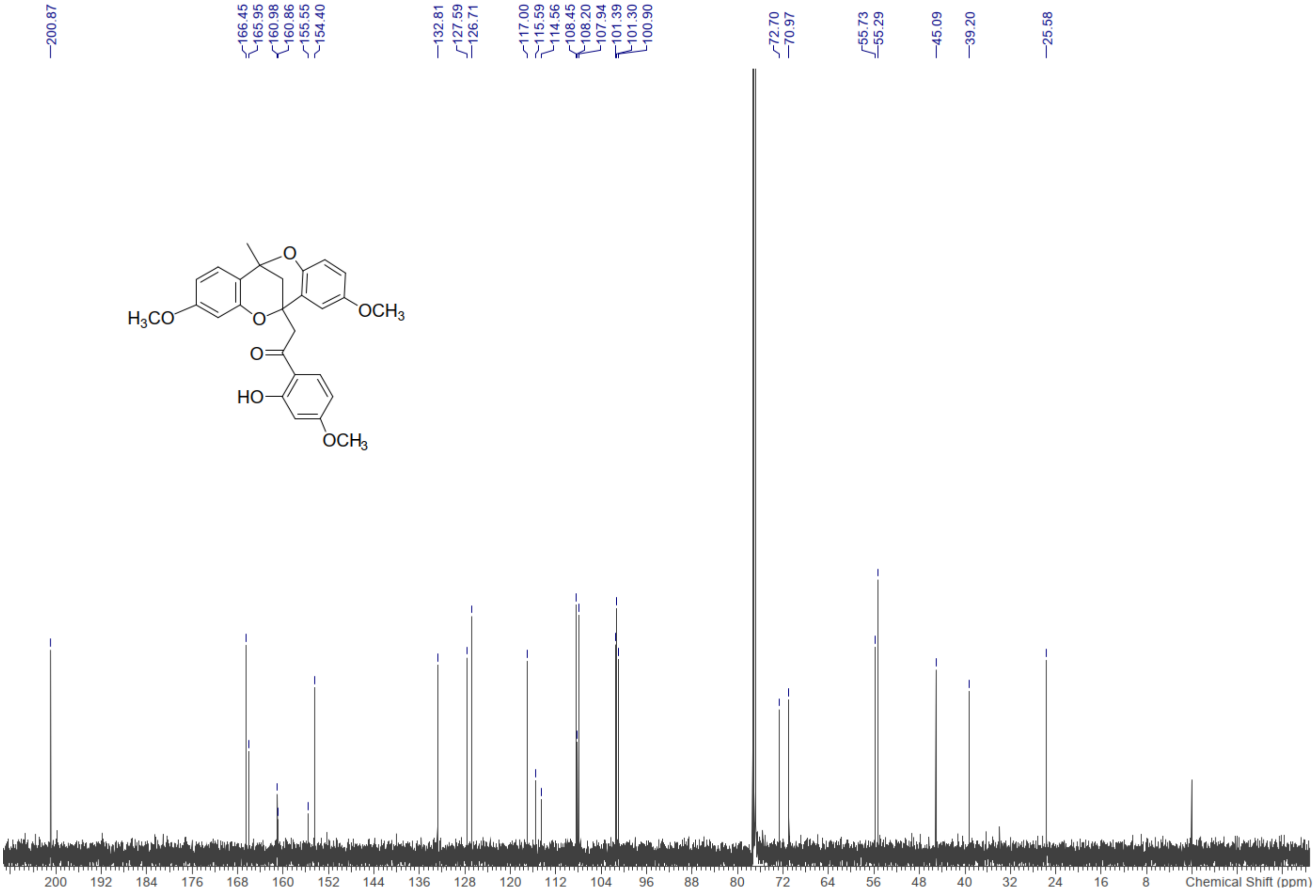

Supplement: Supplementary file 1 [file molecules-24-02405-s001.pdf]
